# Discrimination, Church Support, Personal Mastery, and Psychological Distress Among Black People in the United States

**DOI:** 10.1093/geroni/igab046.1543

**Published:** 2021-12-17

**Authors:** James Muruthi, Bertranna Muruthi, Reid Thompson Cañas, Lindsey Romero, Abiola Taiwo, Peter Ehlinger

**Affiliations:** 1 University of Oregon, Eugene, Oregon, United States; 2 University of Oregon, University of Oregon, Oregon, United States; 3 university of oregon, University of Oregon, Oregon, United States; 4 University of Oregon, 97402, Oregon, United States

## Abstract

Objective: This study used the stress process model to test the mediating effects of personal mastery and moderating effects of church-based social support on the relationship between everyday discrimination and psychological distress across three age groups of African American and Afro-Caribbean adults. Methods: Using a national sample of 5008 African Americans and Afro-Caribbean adults from the National Survey of American Life Study, this study employs structural equation modeling to investigate the relationships between everyday discrimination, personal mastery, church-based social support, and psychological disorders. Results: Everyday discrimination was an independent predictor of psychiatric disorders across all groups. Group- and age-specific comparisons revealed significant differences in the experience of everyday discrimination and psychiatric disorders. Mastery was a partial mediator of the relationship between discimination and psychiatric disorder among Afro-Caribbeans while church support was a significant moderator only among the young and older African Americans. Implications: Together, our study findings provide useful first steps towards developing interventions to reduce the adverse psychological impacts of everyday discrimination on African Americans and Afro-Caribbeans. Intervention efforts such as individual psychotherapy aimed to improve Afro-Caribbean individuals’ sense of mastery would be a partial solution to alleviating the adverse effects of discrimination on their psychological health.

